# Addressing structural restrictive practices in disability support organizations

**DOI:** 10.3389/fpsyt.2026.1790121

**Published:** 2026-05-25

**Authors:** Victoria Sánchez-Gómez, Laura Esteban, Natalia Alvarado, Miguel Ángel Verdugo, Laura García-Domínguez, Blanca Palomero-Sierra

**Affiliations:** 1 Institute for Community Inclusion, University of Salamanca, Salamanca, Spain; 2 Department of Basic Psychology, Psychobiology and Methodology of Behavioral Sciences, University of Salamanca, Salamanca, Spain; 3 Department of Personality, Evaluation and Psychological Treatment, University of Salamanca, Salamanca, Spain

**Keywords:** blanket rules, organizational culture, quality of life, restraint, restrictive practices, structural practices

## Abstract

**Background:**

The Convention on the Rights of Persons with Disabilities emphasizes individual rights and autonomy. However, certain restrictive practices in support services for people with intellectual and developmental disabilities (IDD) may limit these rights and undermine autonomy. Structural practices are a type of restrictive practice usually defined as blanket rules or prohibitions that affect all users equally. They are considered restrictive because constrain the freedom of individuals with disabilities. Since these practices are so deeply ingrained in the organizational culture, they often become normalized and invisible. Despite the implications of structural practices for the rights of people with IDD, empirical evidence on their prevalence is limited, particularly in Spain. In this context, this article aims to analyze the presence of structural practices in different support services for people with IDD in Spain.

**Methods:**

A cross-sectional study was conducted with 45 disability support organizations, which participated by assessing the occurrence of restrictive practices, including structural practices, in their daily contexts using the LibRe scale. Among the structural practices, aspects related to the organization of time and activities, information management and decision-making, money management and established organizational norms were evaluated. The organizations conducted the assessments in teams, involving 194 professionals, 6 family members and 25 individuals with disabilities in the process. The assessment was part of an organizational transformation process. Frequency analyses and ANOVA tests were conducted to identify the most common restrictive practices.

**Results:**

Although organizations generally report a low frequency of restrictive practices, structural practices are significantly more common than others, such as physical or mechanical restraints. Within the structural practices, those referring to limitations in the organization of time and activities of daily living of people with disabilities stood out as frequent practices. Among the most frequent practices were limitations on recreational activities and inflexible meal schedules.

**Discussion:**

Implications of the restrictive practices most frequently reported by organizations on users’ quality of life are discussed, as well as the need to reduce structural practices through organizational transformation approaches. Future lines of research are also highlighted.

## 1 Introduction

The Convention on the Rights of Persons with Disabilities established among its general principles the promotion of individual autonomy, including personal decision-making and independence. Article 19 explicitly states the right of all persons to live independently and make their own choices regarding their way of life, without external constraints (1). For the enjoyment of these rights, the environment in which people live, learn and work is crucial, as it influences their levels of self-determination and the extent to which they have opportunities to choose and exercise control over their lives (2). Several studies have linked less restrictive environments with greater opportunities for decision-making and self-determination (3–10). However, there is still limited understanding of the specific mechanisms within support services that may constrain or facilitate these opportunities in practice.

In this sense, Bigby and Beadle-Brown (11) sought to identify the cultural characteristics in better performing services in which users enjoyed higher quality of life. These authors found that some distinctive features of the culture of these services included: supporting individuals in living the lives they wanted to live, recognizing and respecting users’ preferences, promoting inclusion, participation, dignity and well-being, implementing person-centered practices, and demonstrating flexibility by putting the needs of the person before those of the organization. Similarly, McGill et al. (12) identify participation, choice and control as crucial characteristics of the culture of capable environments, where the goal is to provide high-quality support without any use of restrictive practices.

Restrictive practices formally defined as ‘any type of act or intervention that limits the freedom of movement or rights of a person with a disability’ (13, 14). These practices are particularly prevalent in services supporting individuals with intellectual and developmental disabilities (IDD), especially those who display challenging behavior (15–17). Staff report that these restrictive practices aim to ensure individuals’ well-being and safety (15), yet Didi et al. (18) emphasize that they can undermine individuals’ integrity and well-being and negatively affect support staff.

Among restrictive practices, restraints in all their forms (physical, mechanical, and chemical) have garnered the most concern and received the most attention in literature and in public policy. However, in recent years, various authors have also emphasized the importance of addressing more subtle and normalized forms of restrictions, such as structural practices (19–21). These structural practices, a type of restrictive practice, refers to general rules or prohibitions that affect all users equally (22) and are considered restrictive because they also constrain the freedom of individuals with disabilities, resulting in harm and distress (19–21). Limited opportunities to choose are associated with an increased risk of depressive symptomatology (23). They commonly include depersonalization (removal of personal possessions or restrictions on access to them), rigidity of routine (fixed and unchangeable schedules for daily activities such as meals, hygiene, rest or leisure), and block treatment—all of which are typical characteristics of institutional culture (20, 24, 25). Importantly, several authors warn that these practices may be present not only in large residential settings but also in small community-based settings (26, 27). Despite the growing conceptual recognition of restrictive practices, empirical evidence regarding the presence and frequency of structural practices in support services remains limited, especially in the Spanish context.

Even though some structural practices are mandatory or required by law (such as drug or alcohol policies), most of those implemented on a daily basis (such as restrictions on meals and bedtimes, dietary constraints, lack of choice in daily activities, or restricted access to personal belongings) merely stem from the established culture and norms that govern the daily functioning of an organization or service (14, 20, 28, 29). In practice, restrictions often emerge through environmental contexts, dynamics, organizational culture or daily routines (20, 28–30). In this sense, organizational culture is ‘a slippery concept’, most easily understood as ‘the way we do things around here’ (11, p. 317).

Such cultural frameworks lead certain restrictive practices, especially structural ones, to be routinely employed over extended periods without professionals questioning the necessity of their continuity (15). Consequently, these practices become normalized, which diminishes the willingness to explore more respectful alternatives with the well-being and rights of those supported by these services (15, 31).

Structural practices constitute a significant challenge both because of their impact on rights and their often hidden or invisible nature. Therefore, efforts must be made to ensure that support services for people with IDD are truly capable environments (12) that promote their self-determination and quality of life (2). Key elements for advancing the reduction of restrictive practices are interventions targeting the physical environment as well as organizational culture and leadership (18, 32). To this end, it is essential to identify and address these structural practices, which, as previously noted, may be deeply ingrained in the daily functioning of the organizations, thereby limiting an individual’s control and self-determination (20). Successfully reducing the use of these restrictive practices requires more evidence, which remains limited in this field (18, 31, 33), especially in the Spanish context.

For these reasons, the aim of this article is to analyze the presence of structural practices in different support services for people with IDD in Spain. To further specify this objective, the following research questions were formulated: a) What is the prevalence of structural restrictive practices in support services for people with IDD in Spain? b) Which types of structural restrictive practices are most frequent within these services? c) Are there differences in the frequency of these practices depending on the type of service?

## 2 Methods

### 2.1 Study design

A non-experimental, descriptive, cross-sectional study (34) was conducted to describe the occurrence of restrictive practices through data collected at a single point in time.

### 2.2 Measures

The LibRe scale (21) was used to assess the prevalence of structural practices. This scale was developed in Spain to support organizational transformation processes toward environments that are more respectful of people with disabilities, grounded in evidence-based decision-making (29). Although the LibRe tool can be used to conduct assessments at both the individual and organizational levels, only the organizational-level component was implemented in this study.

This assessment tool is organized into five domains, each of which assesses a type of restrictive practices: (i) Physical or Mechanical Restrictions; (ii) Chemical or Pharmacological Restraint; (iii) Structural Practices; (iv) Relational Practices; and (v) Practices related to Contexts and Supports. Both the first (Physical or Mechanical Restrictions) and third (Structural Practices) domains contain subdomains (21). In line with the research objective, this study focused on the occurrence of structural restrictive practices, that is, on the findings within the third domain and its subdomains. These subdomains are: (i) Organization of Time and Activities (OTA); (ii) Information Management and Decision-Making (IMDM); (iii) Money Management (MM); and (iv) Established Organizational Norms (EON). All items in this domain are listed in more detail below in the Results section (**Table 1**).

**Table 1:** Distribution of frequencies for each item in each subdomain of structural practices.

Item	Item	Does not apply	Never	Very rarely	Quite frequently	Very frequently	Always/ almost always
OTA	27. The amount of leisure or recreational activities the person wishes to undertake is limited for organizational or family reasons	4.44%	6.67%	8.89%	31.11%	22.22%	26.67%
	28. The duration of daily activities is limited without considering the person needs	0.00%	24.44%	28.89%	15.56%	20.00%	11.11%
	29. The person’s involvement in desired educational activities is restricted for organizational or family reasons	26.67%	26.67%	26.67%	6.67%	11.11%	2.22%
	30. A schedule is imposed for food and beverage access	0.00%	13.33%	26.67%	17.78%	8.89%	33.33%
	31. Restricting the times when the person can smoke, even though there are designated smoking areas available	20.00%	40.00%	15.56%	8.89%	8.89%	6.67%
	32. A schedule for personal hygiene is imposed	8.89%	33.33%	13.33%	17.78%	17.78%	8.89%
	33. The time allocated for personal hygiene is limited	8.89%	55.56%	22.22%	6.67%	2.22%	4.44%
	34. A schedule for changing diapers, sanitary towels or tampons, without taking into account the person’s needs is imposed	2.22%	80.00%	13.33%	2.22%	0.00%	2.22%
	35. The person’s involvement in choosing and planning leisure activities is restricted	2.22%	13.33%	28.89%	35.56%	13.33%	6.67%
	36. The person’s involvement in choosing educational activities is restricted	22.22%	28.89%	17.78%	15.56%	11.11%	4.44%
	37. The person’s involvement in their daily routine decisions is limited	4.44%	20.00%	17.78%	11.11%	17.78%	28.89%
	38. Community involvement opportunities is limited for the person	4.44%	31.11%	24.44%	8.89%	24.44%	6.67%
	39. Opportunities for social interactions with others in a satisfactory manner are limited for the person	4.44%	37.78%	13.33%	17.78%	6.67%	20.00%
	40. The person’s opportunities to learn, explore and experience new activities and places are limited	2.22%	26.67%	31.11%	17.78%	13.33%	8.89%
	41. The person’s enjoyment of free time in activities chosen by them is limited	17.78%	24.44%	15.56%	11.11%	17.78%	13.33%
IMDM	42. The person’s privacy is breached regarding personal information	22.22%	31.11%	20.00%	6.67%	8.89%	11.11%
	43. The person’s knowledge and decision-making regarding their medical treatment is limited	15.56%	24.44%	22.22%	11.11%	15.56%	11.11%
	44. The person’s decision-making regarding their sex life is limited	6.67%	28.89%	28.89%	8.89%	8.89%	17.78%
	45. The information provided to the person about daily activities and circumstances is limited	2.22%	31.11%	31.11%	17.78%	15.56%	2.22%
	46. The information given to the person about situations and circumstances related to their life and future is limited	4.44%	24.44%	37.78%	20.00%	11.11%	2.22%
	47. The person is prevented from making choices about aspects of their image	8.89%	24.44%	26.67%	28.89%	11.11%	0.00%
	48. A shaving or hair removal system is imposed on the person	22.22%	28.89%	6.67%	8.89%	24.44%	8.89%
	49. Specific ways of eating or drinking are imposed on the person without clear justification	0.00%	73.33%	17.78%	6.67%	2.22%	0.00%
	50. The person’s decision-making regarding their diet is limited	2.22%	13.33%	28.89%	22.22%	13.33%	20.00%
	51. The person’s choices about when and with whom they spend their time are limited	8.89%	24.44%	33.33%	15.56%	15.56%	2.22%
	52. Involvement in decisions about the support they receive and who provides that support is restricted for the person	8.89%	15.56%	15.56%	31.11%	20.00%	8.89%
MM	53. The person’s money management is restricted	22.22%	17.78%	8.89%	17.78%	15.56%	17.78%
	54. The amount of money the person can access is restricted	31.11%	15.56%	8.89%	17.78%	8.89%	17.78%
	55. The access to and use of personal hygiene products are restricted for the person	11.11%	42.22%	22.22%	6.67%	4.44%	13.33%
EON	56. The person’s access to spaces for daily use is restricted by a lack of access keys	15.56%	28.89%	17.78%	6.67%	8.89%	22.22%
	57. The person’s use of alternative communication systems devices is restricted	6.67%	62.22%	4.44%	17.78%	0.00%	8.89%
	58. The person’s use of new technologies in their free time is restricted	6.67%	35.56%	28.89%	11.11%	8.89%	8.89%
	59. The person’s privacy during showering or bathroom use	0.00%	46.67%	15.56%	15.56%	11.11%	11.11%
	60. Privacy for sexual behavior is restricted for the person	6.67%	57.78%	22.22%	8.89%	0.00%	4.44%
	61. The person’s freedom to leave certain spaces at will is restricted	0.00%	40.00%	22.22%	17.78%	6.67%	13.33%
	62. Contact with family or friends is limited for the person	11.11%	42.22%	24.44%	4.44%	8.89%	8.89%
	63. Having a partner or interacting with certain people is restricted for the person	8.89%	51.11%	24.44%	8.89%	4.44%	2.22%
	64. The person’s involvement in setting rules for the organization of the space where they live is limited	11.11%	24.44%	20.00%	17.78%	17.78%	8.89%

A direct translation of the main content of the item is provided. The examples included in each item in the original tool have been omitted. For further details, see Verdugo et al. (21). OTA, organization of time and activities; IMDM, information management and decision-making; MM, money management; EON, established organizational norms.

The LibRe scale was designed to be completed collaboratively by a group composed of individuals with firsthand knowledge of the daily environment of people with IDD (e.g., caregivers, professionals, families, people with disabilities themselves). Although the tool is not in an easy-to-read format, it includes concrete examples from everyday life that, according to user reports (29), facilitate the participation of people with disabilities in the discussion and response process.

Teams assess how frequently certain practices occur in their organizations by responding to a series of items. Each item is evaluated based on the frequency of these practices, with the following response options: ‘Never’ (0), ‘Very rarely’ (1), ‘Quite frequently’ (2), ‘Very frequently’ (3), and ‘Always/almost always’ (4). For example, Item 30 (from the Structural Practices domain, within the Organization of Time and Activities subdomain) states: ‘*A schedule is imposed for access to food and drink*’. Teams must then assess how frequently this occurs in the daily lives of people within the organization, selecting one of the options listed above. Since the primary purpose of the tool is to foster discussion and change, its scoring method requires the team to agree on a single response. In the event of disagreement among team members, the team should select the response option that implies the greatest level of restriction (21).

Since the score for each domain corresponds to the means of its constituent items, and each item ranges from 0 and 4, each domain retains a theoretical minimum of 0 and a theoretical maximum of 4 with higher scores indicating greater levels of restrictive practices (21).

### 2.3 Participants

Forty-five disability support services organizations from different autonomous communities in Spain participated in the study, assessing the presence of restrictive practices in their daily contexts.

Regarding the types of services where the assessments were conducted, more than half (51.11%) were residences (n = 23). In this study, ‘residences’ refers to larger-scale, institutionally organized residential care settings with 24-hour support, excluding smaller community-based settings. Other services included in the sample were classified as daycare centers (n = 7), occupational centers (n = 8), and ‘other services’ (n = 7), which encompassed a heterogeneous set of settings, including supported housing and special education centers.

### 2.4 Procedure

The data used in this study were not collected directly by the authors but were provided by organizations belonging to the Confederation Plena Inclusión España, which commissioned the present analysis. The tool was applied as part of an organizational transformation process, whereby organizations develop improvement plans to reduce restrictive practices in these settings.

Assessments were conducted by teams comprising 194 professionals, 6 family members and 25 individuals with disabilities. While all teams included professionals, not all included family members or individuals with disabilities. In this context, ‘professionals’ refers to a diverse range of staff members with sufficient knowledge of the day-to-day operations of the organizations, including direct support workers and frontline supervisors. On average, each team was composed of 4.18 professionals (M = 4.18, SD = 2.41, range = 1–10), 0.16 family members (M = 0.16, SD = 0.48, range = 0–2), and 0.56 individuals with disabilities (M = 0.56, SD = 1.65, range = 0–10). All teams agreed to participate voluntarily, with guarantees of individual and organizational confidentiality; additionally, data were anonymized prior to being shared with the research team.

All practices and procedures carried out within the Plena Inclusión España network are governed by an internal Code of Ethics approved by the governing bodies of the participating organizations. This framework establishes binding ethical principles for service provision, organizational conduct, and data management across its member entities (35). Given that the present study relies exclusively on secondary data generated and governed within this system, ethical oversight was ensured through these established institutional mechanisms rather than through a separate application to an external biomedical research ethics committee.

### 2.5 Analysis

Firstly, to identify the most frequent structural practices, the percentage distribution of responses for each item was reported and analyzed (**Table 1**). To operationalize these findings into actionable categories, a hierarchical classification was established *a priori* based on the cumulative prevalence of practices. Specifically, items were categorized as critical if the combined frequency of responses in the upper poles of the scale—ranging from ‘Quite frequently’ to ‘Always/almost always’—exceeded 50%. Based on the principle of absolute majority, this threshold identifies practices with a predominant presence, suggesting they represent systemic features of the services rather than incidental events. Conversely, items were defined as relevant when this cumulative frequency surpassed 30%. This threshold serves as an indicator of a ‘substantial minority’ or significant trend (approximating a tertile distribution). This stratified approach allows for a clear prioritization of the most pervasive issues (critical) while highlighting emerging patterns that represent a frequent risk to users’ autonomy and rights (relevant).

Second, to describe the observed scores, the means (M), standard deviations (SD), range (minimum–maximum), and 95% confidence intervals (CI) were calculated. To determine whether restrictions were significantly more prevalent in specific domains and Structural Practices subdomains, repeated-measures ANOVA with *post-hoc* analyses was performed. The assumption of normality of residuals was met; therefore, parametric tests were used. Sphericity corrections were applied for each analysis. When the assumption of sphericity was violated (Mauchly’s test), results were reported using the Greenhouse–Geisser correction. *Post-hoc* comparisons were adjusted using the Bonferroni correction to control for Type I errors.

Additionally, to explore the differences between service types, one-way ANOVAs were conducted. Assumptions of normality and homoscedasticity were tested. Where normality was not met (only in the Money Management subdomain), the Kruskal–Wallis test was reported. In cases of heteroscedasticity (which occurred only in the Organization of Time and Activities subdomain), Welch’s ANOVA was applied.

For ANOVA effect sizes, partial eta squared (*η*^2^_p_) was reported, with values of approximately.01,.06, and.14 indicating small, medium, and large effects, respectively (36). All analyses were performed with jamovi version 2.3 (37). For *post-hoc* analyses, Cohen’s d was reported as the effect size for each comparison.

## 3 Results

**Table 1** describes the distribution of the responses obtained for each item across the structural practice subdomains.

In the Organization of Time and Activities subdomain, four items were considered critical: i) Item 27, regarding limitations on the number of leisure or recreational activities a person wishes to engage in for organizational or family reasons, with 80.00% of the teams reporting this occurred at least ‘Quite frequently’, with ‘Always/almost always’ as the most frequent response; ii) Item 30, referring to limitations on the duration of daily activities without considering the person’s needs, where more than half of the responses indicated an occurrence of at least ‘Quite frequently’ (60.00%) with ‘Always/almost always’ as the most frequent response; iii) Item 35, regarding restrictions on individuals’ involvement in selecting and planning leisure activities, in which more than half of responses indicated an occurrence of at least ‘Quite frequently’ (55.56%) with the majority concentrated in the ‘Quite frequently’ option; and iv) Item 37, which assesses how often people’s involvement in decisions related to their daily routine is restricted, with over half reporting an occurrence of at least ‘Quite frequently’ (55.32%), with ‘Always/almost always’ as the most common response (27.66%).

Although not classified as critical, seven items in the Organization of Time and Activities subdomain are considered relevant, as at least 30.00% of the responses fell into the ‘Quite frequently’ category. These include Item 28 (‘the duration of daily activities is limited disregarding the person’s needs’), Item 32 (‘a schedule for personal hygiene is imposed’), Item 36 (‘the person’s involvement in choosing educational activities is restricted), and Items 38–41, which relate to interpersonal relationships, involvement in community settings and leisure activities.

In the Information Management and Decision-Making subdomain, two items were identified as critical. Item 50, which assesses how often people’s choices about their own diet are limited, 55.56% of teams reported that this occurs at least ‘Quite frequently’, while 20.00% indicated that it occurs ‘Always/almost always’. Similarly, Item 52, which assesses how often the person’s involvement in decisions regarding the support they receive is restricted, showed that more than a half of responses indicated an occurrence of at least ‘Quite frequently’ (60.00%). Within this subdomain, although not classified as critical, seven items were considered relevant. These include: Item 43 (knowledge and decision-making opportunities regarding their own medical treatment); Item 44 (knowledge and decision-making opportunities regarding their own sex life); Items 45 and 46 (limited information about aspects of their daily life and future); Items 47 and 48 (limitations on people’s choices about their own image); and Item 51 (the person’s opportunities to decide with whom to spend their time and when).

In the Money Management subdomain, one critical item and one relevant item were identified. Item 53, regarding restrictions on the person’s management of their own money, was classified as critical, with more than half of teams reporting this occurring at least ‘Quite frequently’ (51.11%). Item 54, regarding restrictions on the amount of money the person can access, was classified as relevant, with 44.44% of teams reporting this occurring at least ‘Quite frequently’.

Within the Established Organizational Norms subdomain, no critical items were identified, although four items were considered relevant: those related to the use of certain spaces (Items 56 and 61), privacy during showering or bathroom use (Item 59), and opportunities to participate in setting organizational rules (Item 64).

When comparing Structural Practices with other types of restrictive practices (*F* = 50.7; *p* <.001; *η*²_p_ = .274), Structural Practices were reported as significantly more frequent than Physical and Mechanical Restrictions (*t* = 4.82; *p*_Bonferroni_ <.001; *d* = 0.72) and Chemical or Pharmacological Restraint (*t* = 11.384; *p*_Bonferroni_ <.001; *d* = 1.70). However, they did not differ significantly from the Relational Practices or the Practices related to Contexts and Supports domains.

At the descriptive level within the Structural Practices subdomains, the Organization of Time and Activities (M = 1.54; SD = 0.89; range = 0.07–3.07; CI = 1.27–1.80) and Money Management subdomains (M = 1.64; SD = 1.29; range = 0.00–4.00; CI = 1.24–2.05) showed the highest means. These were followed by the Information Management and Decision-Making subdomain (M = 1.42; SD = 0.77; range = 0.00–2.82; CI = 1.19–1.65), while the lowest mean was reported in the Established Organizational Norms subdomain (M = 1.14; SD = 0.87; range = 0.00–3.56; CI = 0.88–1.40). Comparisons among the structural practices subdomains revealed significant differences (*F* = 5.84; *p* <.006; *η*²_p_ = .034) with a small effect size. *Post hoc* analyses indicated significantly lower restrictions in the Established Organizational Norms subdomain compared to Organization of Time and Activities (*t* = -4.05; *p*_Bonferroni_ = .001; *d* = -0.59), IMDM (*t* = -3.39; *p*_Bonferroni_ = .010; *d* = -0.53), and Money Management (*t* = -3.49; *p*_Bonferroni_ = .007; *d* = -0.55) subdomains, which showed greater restrictions and did not differ significantly from one another. In summary, there were significantly lower levels of restriction in the Established Organizational Norms subdomain compared to those related to activities, information and decision-making, and money management, which did not differ significantly from each other.

In terms of the occurrence of structural practices by service type, residences showed greater restrictions than daycare centers, occupational centers, or ‘other’ services at a descriptive level (**Table 2**). This trend was also observed across each subdomain. Despite this pattern, a one-way ANOVA comparing all four service types simultaneously revealed no significant differences (*F* = 2.20, *p* = .076, *η*²_p_ = .139). However, given the small sample size in service types other than residences, a dichotomous comparison was conducted between residences (n = 23) and the non residential facilities (n = 22). In the latter case, significant differences were reported. This analysis revealed significantly higher rates of structural practices in residences than in the non-residential services (*F* = 5.98; *p* = .019, *η*²_p_ = .122), a trend that extended to the Information Management and Decision-Making (*F* = 4.08; *p* = .050; *η*²_p_ = .087), IMDM (*F* = 4.57; *p* = .038; *η*²_p_ = .096), MM (*χ²*_(1)_ = 6.80; *p* = .021), and EON (*F* = 4.91; *p* = .032; *η*²_p_ = .103) subdomains. Overall, although descriptively residences showed higher levels of structural practices across all domains, no significant differences emerged when comparing all service types simultaneously. However, when comparing residences to all other services combined, significantly higher levels of structural practices were observed in residences, a pattern that was consistent across several subdomains.

**Table 2:** Descriptive statistics by service type.

Domain / Subdomain	Type of service	Mean	SD	Min	Max	CI lower	CI upper
Structural Practices	Residences	1.69	0.85	0.11	3.13	1.32	2.06
	Non-residential settings	1.14	0.63	0.21	2.67	0.86	1.42
	*Daycare center*	0.98	0.47	0.42	1.59	0.54	1.42
	*Occupational center*	1.32	0.82	0.34	2.67	0.63	2.01
	*Other*	1.09	0.56	0.21	1.59	0.58	1.61
OTA	Residences	1.79	1.01	0.07	3.07	1.35	2.22
	Non-residential settings	1.27	0.671	0.46	2.85	0.97	1.57
	*Daycare center*	1.02	0.40	0.46	1.67	0.65	1.38
	*Occupational center*	1.46	0.78	0.53	2.85	0.81	2.11
	*Other*	1.31	0.77	0.53	2.78	0.60	2.02
IMDM	Residences	1.65	0.73	0.09	2.82	1.33	1.96
	Non-residential settings	1.18	0.74	0.00	2.55	0.85	1.50
	*Daycare center*	1.13	0.54	0.36	1.91	0.63	1.63
	*Occupational center*	1.34	0.95	0.18	2.55	0.55	2.13
	*Other*	1.05	0.72	0.00	1.71	0.38	1.71
MM	Residences	2.10	1.35	0.00	4.00	1.50	2.7
	Non-residential settings	1.11	1.01	0.00	3.00	0.63	1.60
	*Daycare center*	1.21	0.90	0.00	2.33	0.39	2.04
	*Occupational center*	1.00	1.25	0.00	3.00	-0.04	2.04
	*Other*	1.17	0.88	0.00	2.00	-0.24	2.57
EON	Residences	1.41	0.87	0.00	3.56	1.03	1.78
	Non-residential settings	0.86	0.80	0.00	2.75	0.51	1.21
	*Daycare center*	0.71	0.69	0.17	2.13	0.07	1.35
	*Occupational center*	1.04	0.95	0.33	2.75	0.24	1.83
	*Other*	0.79	0.78	0.00	1.86	0.07	1.51

OTA, organization of time and activities; IMDM, information management and decision-making; MM, money management; EON, established organizational norms.

## 4 Discussion

The aim of this study was to explore the occurrence of structural practices in Spain. The results shed light on the reality of support services for people with IDD in the country. Findings indicate that structural practices are more prevalent than other forms of restrictive practices, which have traditionally received greater attention in the specialized literature (21)–namely, physical, mechanical, or pharmacological restraint. Although more subtle and less visible than other restrictive practices, structural practices equally limit the autonomy and quality of life of service users, according to several authors (19–21). Overall, this remains a topic with limited empirical research.

Among the structural practices analyzed, restrictions related to organization of time and activities were most prevalent, followed by restrictions related to money management and those involving information management and decision-making opportunities. Such practices, widely recognized as a hallmark of life in institutionalized settings (24), conflict directly with the principles of the UN Convention, which upholds the right to individual autonomy and stipulates that individuals should not be forced to live under external constraints (1, 38).

Regarding the organization of time and activities, items such as the imposition of meal and beverage schedules and limited participation in decisions about daily routines and leisure activities reveal a culture where logistical needs take precedence over individual autonomy. This is consistent with previous research showing that organizational policies and routines—and sometimes even staff comfort and preferences—are often prioritized over the individual’s self-determination (39–41). However, it is essential to note that this limitation does not necessarily depend on the will of professionals. Direct support workers often lack adequate resources and face various organizational barriers beyond their control that restrict their ability to offer real choices (42). To manage this discomfort, professionals may sometimes present a decision already made as if it were the person’s own choice (43). This lack of control over daily life also aligns with the findings of Esteban et al. (44), who link the rigidity of routines to group dynamics and the availability of support. Consequently, moving toward personalized support models that guarantee autonomy and independence requires not only a change in attitude within teams, but also the provision and reallocation of resources.

In addition to the limitations on schedules and routines already mentioned, severe restrictions on food choices and meal planning were observed. This finding is consistent with previous research documenting limited food autonomy, particularly in residential settings, where individuals had minimal participation in decision-making and control remained concentrated in the hands of staff (45–47). This dynamic, often associated with infantilizing practices, persists even in certain community-based settings (47, 48). Such practices may stem from concerns about health and physical well-being, and as Karban et al. (49) noted, professionals often struggle to balance safety and self-determination. A shift in mindset is required to recognize that ensuring a person’s well-being also inherently involves respecting their autonomy and independence. Therefore, support should aim to enable individuals to become the causal agents of their own well-being through their own decisions (50, 51).

Of particular relevance are restrictions on decisions about the type of support received and the person providing it. Independent living is not defined by the ability to perform tasks without assistance, but by exercising the agency to choose and manage the support needed. Consequently, this constitutes a central pillar of autonomy for individuals who require significant assistance with daily activities (38, 44).

Rules regarding the management and amount of money available to individuals constitute another way of limiting people’s control over their own lives. This restriction is particularly relevant considering that financial agency plays a pivotal role in decision-making across different areas of life, such as leisure activities or personal possessions. In fact, the availability of money and the provision of supported decision-making in financial matters has been identified by people with IDD as essential requirements for independent living (52).

Although to a lesser extent than the cases mentioned above, restrictions related to personal hygiene routines, the duration of these activities, and the lack of privacy while performing them were also reported with relative frequency. Likewise, limitations in decision-making regarding personal image were identified, contributing to depersonalization, a well-documented characteristic of institutionalized services (24).

In a similar vein, restrictions were observed in decisions related to sexuality, confirming previous findings that indicate that people with IDD rarely have real opportunities to make decisions about these matters, as they face limitations on their privacy and, in some cases, are explicitly prohibited from being alone with their partners (53–55). In addition to these restrictions, individuals face limitations on access to relevant information regarding their health, medical treatments, and other aspects that are significant to their daily lives and future. Such barriers diminish their opportunities to participate in important decisions. Furthermore, participation in leisure activities, community involvement, and interpersonal relationships is often restricted, alongside barriers such as being prevented from leaving a space at will or lacking a key to access daily-use areas. These latter barriers directly undermine the possibility of exercising control over one’s own environment, a central component of self-determination. Taken together, these findings highlight the need to strengthen person-centered planning processes as a key strategy for ensuring that individuals actively participate in decisions that affect their lives, thereby promoting greater control and self-determination (56).

When analyzing differences according to service type, it is evident that restrictions are more common in traditional institutionalized settings, such as large residences. This is consistent with previous studies showing that living in smaller-scale residential arrangements is associated with greater self-determination (57–59). Furthermore, this relationship is mediated by the extent to which the support provided promotes choice and control over people’s own lives (58, 59).

Organizational culture and the nature of support provision influence individuals’ quality of life (11). Although this study does not directly assess the impact of restrictive practices on quality of life, its findings have important theoretical implications for individuals with IDD, as previous research highlights that organizational culture and opportunities for choice can shape well-being and self-determination. Self-determination is a fundamental factor in achieving meaningful personal outcomes (60). Several studies highlight that the ability to choose and exercise control contributes to well-being across multiple dimensions, including social inclusion, interpersonal relationships, satisfaction, and self-esteem (61, 62), a pattern also observed among people without IDD (63).

Building on these findings, several practical implications for service provision can be identified. The LibRe scale can be used by organizations as a self-assessment tool to identify and reflect on structural restrictive practices that are often normalized in daily routines. Beyond raising awareness, it enables services to detect specific areas where autonomy is limited and to develop targeted improvement strategies.

In practice, organizations could use these results to evaluate their everyday practices, identify priority areas such as the organization of time, financial management, and decision-making, and progressively introduce changes aimed at reducing these restrictions. This may include increasing flexibility in daily routines, ensuring genuine opportunities for choice, promoting supported decision-making, and enhancing individuals’ control over their finances.

More broadly, these changes require a shift from organization-centered models towards person-centered approaches grounded in human rights, involving both changes in staff practices and organizational adjustments to support greater autonomy (44, 45, 64).

In addition to these practical implications, several strengths of this study are worth noting. The primary strength lies in bringing visibility to practices that are deeply rooted in organizational culture, making them widely established, normalized, and rarely analyzed empirically. Furthermore, the use of the LibRe scale, designed specifically for the Spanish context and oriented toward organizational transformation processes, enables the identification of restrictive practices through an approach aligned with human rights and quality of life. Likewise, the participation of multiple organizations and the comparison across different types of services provide an ecological perspective on the phenomenon, reinforcing the applied relevance of these findings.

Beyond the strengths and implications of this study, certain limitations must be acknowledged. Primarily, because the data rely on organizational self-assessments, there is a risk of social desirability bias. This may lead to an underestimation of the actual occurrence of certain practices, particularly those deeply entrenched within the institutional culture. In this sense, the limited involvement of families and individuals with IDD in evaluation processes, compared to the number of professionals, may contribute to this bias. Including the perspectives of people with disabilities and their families is essential to ensure that decisions and interventions truly reflect their needs and preferences. This is particularly crucial in processes aimed at enhancing individual’s control over their own lives. Another limitation of the study is the small number of non-residential settings included in the sample, which may have influenced the results and constrained insights into the implementation of practices in these settings. Finally, the cross-sectional and descriptive design prevents the establishment of causal relationships or the analysis of changes over time. Given these limitations, future research should adopt longitudinal designs that allow the evaluation of specific intervention strategies aimed at reducing such restrictions across diverse support contexts. Studies should also expand sample sizes and further examine the organizational and cultural factors underpinning these practices. As research in this area remains at an early stage, additional organizational data are needed to better understand the scope, distribution, and interrelationships of structural restrictive practices across services. This evidence is essential to strengthen the empirical foundation of a rights-based approach to supporting people with IDD.

## Data Availability

The data analyzed in this study is subject to the following licenses/restrictions: The data were collect by external evaluators (Plena Inclusión España) and provided to the authors in anonymized form. Due to ethical and privacy considerations the data set is not publicly available. Access may be granted upon reasonable request and with permission from the data providers. Requests to access these datasets should be directed to Laura Esteban, lauraestesa@usal.es.
